# The Effect of Preventive Agents (Mouthwashes/Gels) on the Color Stability of Dental Resin-Based Composite Materials

**DOI:** 10.3390/dj5020018

**Published:** 2017-06-15

**Authors:** Khalid H. Al-Samadani

**Affiliations:** Department of Restorative Dental Science, College of Dentistry, Taibah University, Al Madinah Al Munawwarah 43353, Saudi Arabia; kalsamadani@gmail.com; Tel.: +966-557-67-6802

**Keywords:** composite resin, Flocare gel, aesthetic restoration, preventive mouthwashes

## Abstract

The color of dental restorative material should be maintained throughout its functional lifetime in an oral environment. However, the frequent use of mouthwash may affect the color stability of these composite restorations. The aim of this study is to assess the effects of using various mouthwashes on the color stability of various dental restorative composite materials. For this purpose, four mouthwashes/gels (Flocare gel (0.4% stannous fluoride), Pascal gel (topical APF fluoride), Pro-Relief mouthwash (sodium fluoride), and Plax Soin mouthwash (sodium fluoride)), and distilled water as a control, were selected. These were divided into five groups: Group 1: Flocare gel; Group 2: Pascal gel; Group 3: Pro-Relief mouthwash; Group 4: Plax Soin mouthwash; and Group 5: distilled water (control). Prepared restorative materials samples were immersed in the groups of mouthwashes/gels and the distilled water (control) for 24, 48, and 72 h. The discoloration that all materials exhibited with all immersion groups was significantly different at each of the three time periods for all groups (*p* < 0.05). Results from immersion in Flocare gel, Pascal gel, Pro-Relief mouthwash, and Plax Soin mouthwash were statistically significant (*p* < 0.05). The color change chroma was not significant for Pro-Relief and Plax Soin mouthwash (*p* > 0.05). Mouthwashes/gels affect color shifting for all composite resin materials, and changes are exaggerated over time. However, discoloration effects are not perceptible to the human eye.

## 1. Introduction

Patient awareness of dental aesthetics has been increasing for several years. Therefore, the demand for aesthetic restoration depends upon the reproduction of tooth shape and shade, as well as on maintaining the selected color throughout the restoration’s functional lifetime in the oral environment [1]. Recently introduced tooth-colored restorative material has been widely used to restore cavitated lesions in order to satisfy patients’ aesthetic demands [2]. However, after restorations, the frequent use of mouthwash could affect the color stability of these composite resin restorations [3].

Color translucency and opalescence are optical properties required for natural appearance [4,5]. The clinical performance of dental resin composites has been significantly improved in recent decades through innovations using nano-fillers to produce adequate strength and excellent wear resistance along with retaining translucency [2,6]. It is generally believed that a reduced filler size will increase resistance to discoloration, but one recent study showed that filler size did not affect color stability [7]. Moreover, hybrid resin composites have better color stability compared to nano-filled composites [8,9]. Discoloration of composite resin filling materials may be caused by several intrinsic and extrinsic factors [10]. Discoloration of composite resin restorations may occur from time to time, and these color changes may lead to replacing restorations due to their unacceptability to patients [11]. Extrinsic factors causing discoloration of resin composites include staining by adsorption or absorption of colorants from exogenous sources such as coffee, tea nicotine, beverages, and mouthwash [12]. In addition, the physical properties of finished composite materials (such as surface roughness) may influence staining behaviors [13].

In order to prevent dental caries and periodontal diseases, a number of preventive remedies, including fluoride application [14,15] and bactericidal agents such as chlorhexidine [16,17,18], are commonly used. Antimicrobial mouthwashes are used to control dental plaque, dental caries, and periodontal disease [9,19]. However, the frequent use of mouthwash may have adverse effects, although, despite the increased use of mouthwash, researchers have reported a limited effect on color changes of the composite resin [20]. Tooth-colored restoration materials are widely used in dental practice to meet patients’ aesthetic demands. Different types of composite resin with different physical characteristics are available on the market. They are classified by particle size, shape, and distribution of fillers [21]. Filler size has been progressively reduced to provide smoother surface textures that in turn result in optimized optical properties [22,23]. Ideally, restorative materials should not change in color or appearance, but a degree of color change can be caused by a number of factors, including incomplete polymerization, water sorption, chemical reactivity, oral hygiene, and the surface smoothness of the restoration [24]. Furthermore, saliva, food components, different drinks, beverages, and mouthwashes may affect the color of provisional restorations. The aim of this study is to assess the effects of using various mouthwashes/gels and mouthwashes on the color stability of various dental restorative composite materials. This study compares five different resin composite materials for total color difference for various aging times.

## 2. Results

**Figure 1:**
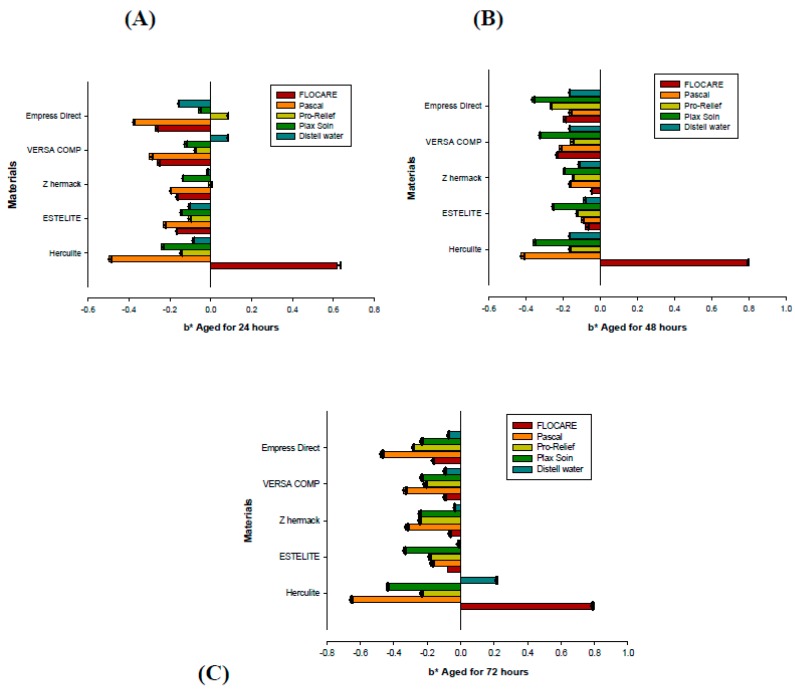
The color difference (Δb*) of the composite resin materials in response to various preventive anti-caries gels and mouth rinse solutions after aging: (**A**) 24 h; (**B**) 48 h; (**C**) 72 h.

Color shifts from the effects of preventive mouthwashes and gel on five types of restorative materials, with different immersion times, were observed in the present study. The effects of immersion duration and different mouthwashes/gels and gel types—Flocare gel (0.4% stannous fluoride), Pascal gel (topical APF fluoride), Pro-Relief mouthwash (Na fluoride), and Plax Soin mouthwash (Na fluoride)—were assessed, focusing on the mean values of color stability in terms of color changes ΔE*_ab_ (Table 1) and Δb* for all materials (Figure 1), after aging for 24 h, 48 h, and 72 h.

Total color differences ΔE*_ab_ and Δb* for the materials tested were analyzed statistically using one-way analysis of variance (ANOVA) and the Tukey’s test (*p* < 0.05). There were significant differences (*p* = 0.05) in color changes between materials tested at the three different immersing periods for each group, as seen in Table 1. Color stability for the five tested composite resin materials, comparing the initial values before immersion to the post-immersion (final measurement) ΔE*_ab_, shows that the materials were discolored. In addition, the color changes exhibited in all groups for the tested materials after time elapsed (24, 48, and 72 h) were significantly different. The total color difference ΔE*_ab_ shows the discoloration of all materials included in this study.

For the materials in all groups, the total color differences, ΔE*_ab_, increased as immersion time increased, with some exceptions. For Group 1 (Flocare gel), all the materials’ discoloration increased as time elapsed, except that the Versa Comp Sultan material discoloration did not change between 24 and 72 h (*p* = 0.00), a highly significant finding. For Group 2 (Pascal gel), the ΔE*_ab_ increased with the time interval except for Estelite Ʃ Quick, Versa Comp Sultan, and Empress Direct IPS, all of which showed decreased discoloration at 72 h compared to that at 48 h (*p* = 0.001), again a highly significant result. Group 3 (Pro-Relief mouthwash) showed significant discoloration of all materials, increasing as immersion time increased (*p* = 0.042). For Group 4 (Plax Soin mouthwash), all materials’ discoloration increased as time elapsed, except for Versa Comp Sultan and Empress Direct IPS, which showed significantly decreased discoloration at 72 h (*p* = 0.004). Group 5 (the control) also showed significant increasing discoloration with time (*p* = 0.008). After 24 h of immersion, the highest ΔE*_ab_ was that of the Empress Direct IPS in the Flocare gel group, and the lowest was Versa Comp Sultan in the Pro-Relief mouthwash group. At the 48 h point, the highest and the lowest ΔE*_ab_ were in Group 4 (Plax Soin mouthwash), where the highest value was that of the Empress Direct IPS and the lowest was the Zhermack material. After 72 h of immersion, the highest ΔE*_ab_ was that of the Empress Direct IPS in Group 3, the Pro-Relief mouthwash, and the lowest was for the Herculite XRV Ultra in Group 4, the Plax Soin mouthwash.

The color changes (Δb*) in Group 1, aged with Flocare gel, for 24, 48, and 72 h was imperceptible to the human eye, since Δb* < 1.5 (clinically acceptable). All materials showed discoloration, and color shifting increased as immersion periods increased, decreasing in blueness, except for the Herculite ^TM^ XRV Ultra material, which showed increased discoloration towards the yellow axis as time elapsed; however, all changes were clinically acceptable. The color changes showed statistically significant values (*p* = 0.000). The total color difference changes in Group 2, the Pascal anti-caries gel (APF preventive gel), for periods of 24, 48, and 72 h, were imperceptible to the human eye; that is, ΔE*_ab_ < 1.5 for all tested materials (clinically acceptable). All materials showed increases in ΔE*_ab_ towards the yellow axis with time elapsed, and the highest total color difference was for Empress Direct IPS 1.12, although materials of Estelite Ʃ Quick and Versa Comp Sultan decreased in yellowness after 72 h of aging, a statistically significant result (*p* = 0.001). (Figure 1).

The Δb* in Group 2 (Pascal anti-caries gel (APF preventive gel)), was imperceptible to the human eye at all aging times, where Δb* < 1.5 (clinically acceptable). The color shifting for this group was towards the blue axis; all materials at 48 h of immersion showed a decrease in their blueness values compared to 24 h; at 72 h of immersion, the shifting of the color for all materials was highly increased, again towards blueness. The shift was statistically significant (*p* = 0.016), but imperceptible to the human eye.

For Group 3 (Pro-Relief mouthwash (Na fluoride)) total color difference ΔE*_ab_ of the composite resin materials tested increased significantly, with differences in the periods of 24, 48, and 72 h (*p* = 0.042). The discoloration of the materials was towards the yellow axis, with increasing yellowness of the materials as periods of immersion increased. Color shifts were imperceptible to the human eye in all the periods, with ΔE*_ab_ < 1.5, and clinically acceptable, except that of Empress Direct IPS, which was perceivable to the human eye, with ΔE*_ab_ > 1.5 (1.65) at 72 h of immersion, which is not clinically acceptable. In this group, the color shifting of Δb* for the materials aged for 24, 48, and 72 h was not significantly different (*p* = 0.794); it increased towards the blue axis with time elapsed, but was imperceptible to the human eye in all aging periods Δb* < 1.5 (clinically acceptable), again except the Empress Direct IPS material. Empress Direct IPS discolored at 24 h towards yellowness; however, after 48 h of immersion, the color of the material shifted from the yellow axis direction, towards blueness, and kept increasing towards the blue axis as immersion time increased.

For Group 4 (Plax Soin mouthwash (Na fluoride)), the total color difference ΔE*_ab_ of the composite resin materials tested showed an increase in discoloration with respect to elapsed time, i.e., towards greater yellowness. Discoloration increased significantly (*p* = 0.004) from 24 to 48 to 72 h, except for the Versa Comp Sultan and Empress Direct IPS materials, where the color changes decreased at 72 h. Color change was imperceptible to the human eye and was clinically acceptable, at ΔE*_ab_ < 1.5. The exception was the Empress Direct IPS, which, at 48 h of immersion, had ΔE*_ab_ > 1.5 (1.95); the color shift was perceivable to the human eye and is clinically unacceptable and statistically significant (*p* = 0.004). Color changes of the Δb* values in Group 4 increased across 24, 48, and 72 h towards the blue axis and were imperceptible to the human eye, with clinically acceptable values of Δb* < 1.5. The materials Versa Comp Sultan and Empress Direct IPS decreased in blueness at 72 h of immersion, though the change was not statistically significant (*p* = 0.513).

Group 5 (the distilled water control) exhibited color differences towards yellowness, increasing with aging (*p* = 0.008). The Δb* chroma value showed no significant differences between materials in the color changes (*p* = 0.189), with all materials discolored towards the blue axis and increasing with time to 48 h; however, all materials shifted back by decreasing in blueness color change at 72 h, except for Herculite XRV Ultra, which showed a shift in color from blue to yellow (Figure 1).

## 3. Discussion

The main reason for the replacement of aesthetic restorations is the color instability of composite materials [9]. The color changes of the materials can be assessed using various instruments, including spectrophotometers and colorimeters [9,25]. The Commission International de Eclairage (CIE) Lab system is used to record minor color differences [26]. The color changes in composite resin are associated with factors such as composition, resin matrix, dimension of fillers, coloring agents, and polymerization depth [27]. The eye can detect changes in Δb* values greater than 1.5. The Δb* value represents the yellow–blue axis, where any changes in b* value reveal important and significant color shifts, since the visible-light-cured composite resin contains yellow from the photo initiator.

This study evaluates the effect of four commercially available preventive agents, using distilled water as a control, on the color stability of five resin materials having different compositions of hybrid, micro-hybrid, and nano-hybrid resin-based materials. The mouthwashes and gels contain different types of fluoride as preventive (anti-decay and -caries) agents, and the resins were treated by immersion in the preventive mouthwash rinses and gels, with different intervals of time elapsed: 24 h, 48 h, and 72 h. We calculated these times to be equivalent to years of daily mouthwash use, where two minutes of use daily for two years equals 24 h [28,29]. Therefore, the immersion time in the groups within this study thus represents two years, four years, and six years of daily use of each agent. All the mouthwashes/gels used within this study contain different types of fluoride particles as preventive ingredients: 0.4 stannous fluoride (Flocare gel), APF fluoride (Pascal gel), and Na fluoride (Pro-Relief mouthwash and Plax Soin mouthwash). The fluoride particles have adverse effects on the resin matrix of the materials, the monomers content in the resin matrix [5]. Daily use of these agents may increase discoloration of the composite materials. However, the measured color differences were imperceptible to the human eye.

Decay leads to chemical softening and affects the surface degradation and rigidity of the materials, leading to increases in the surface roughness of composite resin restorations [29]. Rough surface mechanically retain stains more than smooth surfaces [30,31]. In terms of visual evaluation, discoloration can be considered acceptable up to the value ΔE*_ab_ = 3.3, which is considered the upper limit of acceptability [30]. Color variations (ΔE*_ab_) were calculated between the three color positions at 24 h, 48 h, and 72 h of immersion; in 3-D, L*, a*, and b* color space was evaluated. The mean values of color change for all tested materials after exposure to all mouthwashes and gels, and to the distilled water control, at 24, 48, and 72 h of immersion are summarized in Table 1 and Figure 1. The discoloration that all materials exhibited in all immersion groups was significantly different at each of the three time periods for all groups (*p* < 0.05). Total color difference values of 3.3 or greater were considered clinically perceptible based on previous findings, where ΔE* values less than 1 were regarded as imperceptible to the human eye, since the human eye cannot detect ΔE* values of less than 1.5. Values were calculated using the colorimeter [9,32].

The color differences of composite resin materials tested in this study were imperceptible and clinically acceptable when immersed in the different mouthwashes and gels and the control group of distilled water for 24, 48, and 72 h, except for Empress Direct IPS, where discoloration after 48 h immersed in the Plax Soin group was 1.95, more than the 1.5 value that is detectable according to previous studies [9]. Total color differences were imperceptible and clinically acceptable when the aesthetic composite resin materials were immersed in distilled water (control group) for 24, 48, and 72 h, and this confirms that water sorption does not alter the composite color [9]. The effects of mouthwashes and gels on color stability are no different from distilled water (because the mouthwash contains water in its composition in addition to fluoride particles as a preventive ingredients, which may have an adverse effect on color shifting), although visually imperceptible discoloration did occur after the immersion periods. Significant discoloration occurred in each group of mouthwashes/gels over time, where the color shift in the different groups was significantly different when total color difference within the tested groups was compared after 24, 48, and 72 h of immersion (*p* < 0.05). The mouthwash and gels affected the color of the specimens over the 72 h period (*p* < 0.05).

In previous reports, it has been demonstrated that minimum differences in the CIE LAB color parameters ΔL*, Δa*, and Δb* of 1 CIE unit is perceptible to an observer based on the background color, CIE L* value, and lighting, where the differences in the range of 1–2 CIE units are usually noticeable [9,33]. According to this study’s findings, discoloration in Group 1, (Flocare gel (0.4% stannous fluoride)), aged for 24, 48, and 72 h was imperceptible to the human eye: ΔE*_ab_ < 1.5. The highest discoloration was to Empress Direct, which showed an IPS ΔE*_ab_ = 1.39 after 72 h, a clinically acceptable result. The Flocare gel preventive agent group presented the most statistically significant values (*p* = 0.000).

The b* values represent the chroma value from (+) yellowness to (−) blueness, and the Δb* value is the color change along the blue-yellow axis. The value of b* is derived from b* = bf* − bi*. The value of bf* is the final measurement after aging, and bi* is the initial measurement before aging. (Figure 1). For Group 1 (Flocare gel (0.4% stannous fluoride)), the Δb* value showing discoloration of the materials was highly significant (*p* = 0.000), especially for the Herculite XRV Ultra (BIS GMA) material, which showed an increase in discoloration towards yellowness with an increased immersion time. For all the other materials, discoloration decreased towards blueness over time, except for Estelite Ʃ Quick (bismethacrylate (10%–30%), ethylenedioxydiethyl dimethacrylate), for which the decrease towards blueness showed the same values after 48 h, and for Zhermack (EBDADMA, TEGDEMA), which decreased after 48 h of immersion and then increased after 72 h of immersion. For Group 2 (Pascal gel (APF fluoride)), the Δb* discoloration was significant (*p* = 0.016). The discoloration of all materials was towards blueness, where all materials at 48 h decreased in color, changing towards blueness, while all materials after 72 h of immersion shifted again, increasing more towards blueness.

Group 3 (Pro-Relief mouthwash (Na fluoride)) showed no significant difference between the color change of the materials (*p* = 0.794), whereas all other materials increased discoloration blueness except for Empress Direct IPS (dimethacrylates 20–21.5 wt %), which discolored towards yellowness after 24 h, but after 48 h of immersion, the materials’ color shifted from yellowness to blueness, and blueness increased as immersion time increased. Group 4 (Plax Soin mouthwash (Na fluoride)) showed no significant differences in discoloration of the materials (*p* = 0.513). The materials exhibit more discoloration towards blueness as immersion time increased, except for Versa Comp Sultan (BIS-GMA) and Empress Direct IPS (dimethacrylates 20–21.5 wt %), which, at 72 h of immersion, shifted back, decreasing in blueness. Group 5 (the control of distilled water) shows significant discoloration towards the yellow axis, increasing gradually as time elapsed (*p* = 0.008). The Δb* value shows no significant differences in color change (*p* = 0.189) between materials; all materials discolored towards blueness, increasing over time to 48 h, but all materials shift back in decreasing blueness at 72 h of immersion, except for Herculite XRV Ultra (BIS GMA), which shows color shifting from blueness to yellowness (Figure 1).

The results and findings in this study in agreement with previous studies on the adverse effects of preventive ingredients of the aging mouthwash solution of the Na fluoride on the resin matrix of composite materials (clinically unacceptable) [23,29]. Immersion periods in this study were increased up to 72 h (equivalent to 6 years), according to the suggestions of the previous study stated that the future studies should consider longer periods of immersion [10].

The different preventive anti-caries and -decay gels and solutions, with different preventive ingredients of stannous fluoride, APF, and Na fluoride, showed color shifting in all materials. They caused considerable discoloration in the composite resin materials with periods of 24, 48, and 72 h of aging time. A significantly different change in total color difference ΔE*_ab_ and Δb* values appeared for all tested materials in the Flocare gel and Pascal gel groups with 24, 48, and 72 h of aging time. The duration of aging does have an effect on the color stability of composite resins; an increasing immersion time leads to more intense color changes.

These shifts are due to an increased interaction between the components of the preventive ingredients and their penetration into the resin [33]. Previous studies have shown that the effects of mouthwashes on the color stability are no different from those of distilled water; however, although visually imperceptible, mouthwashes do affect color stability. The water component of the mouthwashes might also affect the color shift changes [30]. The potential effects of aging mouthwash solutions have been reported. The effects of aging solutions on the color stability of resin composite materials have mainly depended on factors such as solution type, exposure time, and chemistry. The composition of the composite resin materials including the resin formulations (the type of photo initiator) and filler size has a direct impact on the staining of the material by external agents. Extrinsic factors for discoloration include staining by adsorption or absorption of colorants as a result of staining from exogenous sources [9]. Extrinsic factors for discoloration are known to cause staining of restorations in combination with aging factors, which have been reported with adverse effects of discoloration [9,34]. Moreover, other studies have demonstrated that the low pH of active preventive ingredients of mouthwashes such as fluoride may influence hardness, wear, and color stability [29,35]. The acidity of the preventive solution may change the polymeric matrixes of resin composites affecting di-methacrylate monomers, present in their compositions [29,36]. The previous studies suggested that, by lowering the solution’s pH level, there is a production of methacrylic acid that results in sorption and hygroscopic expansion as a consequence of enzymatic hydrolysis and biodegradation [29,36]. According to previous findings, alcoholic ingredients, in addition to alcoholic substances in the aging solution, are not the only factor causing softening of resin composite materials; mouthwash can contain other substances such as emulsifiers and organic acids, which can lead to surface degradation of the resin composite materials [23,37]. Additionally, in the clinical environment, the aesthetic effects of mouthwashes and gels on composite resin materials depends on many factors that cannot be simulated in vitro; saliva, foods, and beverages, for example, may affect color stability.

## 4. Materials and Methods

### 4.1. Study Design

Four preventive mouthwashes and gels, and distilled water as a control, were studied for their effects on composite resin restoration materials. The solutions were divided into five groups: Group 1: Flocare gel (0.4% stannous fluoride); Group 2: Pascal gel (topical APF fluoride); Group 3: Pro-Relief mouthwash (Na fluoride); Group 4: Plax Soin mouthwash (Na fluoride); and Group 5: distilled water (control). The color stability of five composite resin restorative materials—Herculite XRV Ultra, Estelite Ʃ Quick resin-based composite, Zhermack micro-hybrid composite, Versa Comp Sultan, and Empress Direct IPS resin composite—was evaluated for each study group (Table 2).

### 4.2. Specimen Preparation

A total of 125 specimens of the aesthetic restorative materials were prepared and randomly distributed for each study group (*n* = 30) and control group (*n* = 5). The preparation of the specimens was completed using Teflon molds and light curing, following the manufacturer’s instructions. Each Teflon mold was placed on a glass microscope slide and overlaid with a 22 × 22 mm cover glass (BDH Borosilicate glass) placed at each open end of the mold to act as a separator. The function of the glass slide was to provide compaction of the materials into a flat surface and to act as a separator. The step-wise method of layering was used for the preparation of the composite resin dental restorative filling material specimens, and the compacting of the material was made with a plastic spatula, after which the restorative filling material was irradiated with a 20 s pulse from a light-curing unit light emitting diode (LED) unit (3M ESPE Dental Products D-82229 Seefeld, Germany). The mold was completely filled with material using this step-wise method and was irradiated at each stage with 20 s light pulses according to the manufacturer’s instructions. The final specimen dimensions were discs with an 8 mm internal diameter and a 3 mm height. The specimens were polished with 3M Sof-Lex discs to obtain a clinical finish and stored in distilled water for 24 h at 37 °C before initial color assessment.

### 4.3. Immersion of the Specimens

Standard immersion solutions were prepared for all groups. The specimens were immersed in the preventive mouthwash agents and distilled water (control) at 37 °C, in different groups, to evaluate the color stability of the different tested composite resin restoration materials for periods of 24, 48, and 72 h, which is equivalent to 2 min of mouthwash per day for two, four, and six years. Twelve hours of immersion simulate one year of rinsing [28]. Throughout the study, all specimens in the groups were shaken on an orbital rotational table every 3 h to provide homogeneity.

After thoroughly rinsing each specimen for 120 s with tap water, all specimens were stored in distilled water in a wide mouth dark bottle. The baseline colors for all specimens were calculated using a colorimeter (Minolta CR; Minolta Co., Osaka, Japan) using Commission International de Eclairage (CIE) [9,38] L*, a*, and b* relative to standard illumination A; therefore, a white background was used, and color differences were tested. The colorimeter was calibrated following the manufacturer’s instructions prior to the measurement session, using a white calibration standard. The L* value refers to lightness, which ranges from zero for black to 100 for white, while the chromaticity values are a* and b*, coordinates in the axis of red–green and yellow–blue, respectively. Positive b* values are in the range of yellow and negative values indicate blue. Positive a* values means the color shifts towards red, and the negative values mean the color shifts towards blueness. After 24, 48, and 72 h, all specimens were removed from the immersion solution and flushed with running tap water. Excess fluid on the surface of the specimens was removed gently with tissue papers. After color measurements at each time interval, all specimens in each group were re-immersed in fresh solution. The quantitative evaluation of color difference with a colorimeter confers advantages such as repeatability, sensitivity, and objectivity, despite some limitations. Discoloration can be evaluated with different instruments and techniques.

For chromatic differences assessment, the CIE L*, a*, b* system was used. According to this system, L* represents the lightness of the sample, a* describes the green–red axis [−a = green; +a = red], and b* describes the blue–yellow axis [−b = blue; +b = yellow]. It is also possible to calculate the total color changes [ΔE*_ab_], which considers changes in L*, a* and b*. Variable thresholds of color difference values were reported that are perceptible to the human eye. However, for dental restorative materials, the clinically acceptable value for color changes is set at ΔE*_ab_ ≤ 3.3 [39,40].

The color measurements were repeated five times for each specimen: before immersion (baseline) and after the elapsed immersion times of 24, 48, and 72 h. The color differences ΔE*_ab_ were computed from the mean values of ΔL*, Δa*, and Δb* for each specimens of the tested composite resin material in all groups, using the following equation [5,9,41]:

ΔE*_ab_ = [(ΔL*)^2^ + (Δa*)^2^ + (Δb*)^2^]^1⁄2^.

The ΔL*, Δa*, and Δb* values are differences in the L*, a*, and b* values from baseline after immersion for each interval time (24, 48, and 72 h).

The mean calculated data of the specimens of each composite resin material after immersion with the preventive mouthwashes and gels, as well as of the control groups, at different elapsed times, were statistically analyzed. The mean values of ΔE*_ab_ and Δb* for the materials at different immersing times were compared statistically by one-way analysis of variance (ANOVA) followed with the multiple comparisons test and the Tukey’s test. The significance level was considered as *p* < 0.05.

## 5. Conclusions

All the mouthwashes/gels used in this study affected the color shifting of all composite resin materials, which increased with aging time. The total color difference ΔE*_ab_ was significant for the discoloration of all materials within all preventive agent groups, but changes were not visually perceptible except for the Empress Direct IPS material in the Pro-Relief and Plax Soin mouthwashes, where changes were perceptible and clinically unacceptable due to sodium fluoride (Groups 3 and 4). However, the stannous fluoride and APF (Groups 1 and 2) resulted in discoloration effects that may not be perceptible and are therefore clinically acceptable. The Δb* value for all materials aged with mouthwashes/gels discolored towards blueness, except for Herculite XRV Ultra, which discolored towards yellowness, and the Empress Direct IPS material, which in in Pro-Relief preventive agent initially discolored towards the yellow axis but, with more immersion time, shifted from the yellow to the blue axis, showing adverse effects from sodium fluoride.

## Figures and Tables

**Table 1:** The total color difference (ΔE*_ab_) for tested composite resin materials immersed in mouthwashes/gels (Flocare anti-caries gel, Pascal anti-caries gel, Pro-Relief mouthwash, Plax Soin mouthwash) and the control group, for different aging periods.

Materials	24 h	48 h	72 h	Significance
Flocare anti-caries gel (Group 1) Mean (St.Dev)				
Herculite ^TM^ Xrv Ultra	0.69 (0.031)	0.81 (0.047)	0.84(0.038)	
Estelite Ʃ Quick	0.58 (0.025)	0.53 (0.030)	0.64 (0.015)	
Z Hermack	0.49 (0.010)	0.45 (0.010)	0.63 (0.025)	
Versa Comp Sultan	0.73 (0.025)	0.76 (0.036)	0.76 (0.025)	
Empress^®^ Direct IPS	1.33 (0.025)	1.27 (0.020)	1.39 (0.015)	*p* = 0.000
Pascal anti-caries gel (Group 2)				
Herculite ^TM^ Xrv Ultra	0.98 (0.049)	0.81 (0.031)	1.06 (0.040)	
Estelite Ʃ Quick	0.58 (0.021)	0.64 (0.031)	0.60 (0.015)	
Z Hermack	0.34 (0.026)	0.45 (0.015)	0.70 (0.029)	
Versa Comp Sultan	0.84 (0.021)	0.87 (0.020)	0.75 (0.025)	
Empress^®^ Direct IPS	1.04 (0.020)	0.94 (0.031)	1.12 (0.045)	*p* = 0.001
Pro-Relief mouthwash (Group 3)				
Herculite ^TM^ Xrv Ultra	0.70 (0.015)	0.62 (0.025)	0.99 (0.010)	
Estelite Ʃ Quick	0.37(0.015)	0.47 (0.015)	0.83 (0.015)	
Z Hermack	0.38 (0.015)	0.56 (0.032)	0.89 (0.015)	
Versa Comp Sultan	0.21 (0.031)	0.52 (0.025)	0.83 (0.025)	
Empress^®^ Direct IPS	0.89 (0.021)	1.33 (0.010)	1.65 (0.026)	*p* = 0.042
Plax Soin mouthwash (Group 4)				
Herculite ^TM^ Xrv Ultra	0.25 (0.015)	0.41 (0.021)	0.54 (0.010)	
Estelite Ʃ Quick	0.44 (0.010)	0.50 (0.015)	0.63 (0.021)	
Z Hermack	0.38 (0.010)	0.38 (0.006)	0.68 (0.010)	
Versa Comp Sultan	0.40 (0.015)	1.01 (0.026)	0.69 (0.010)	
Empress^®^ Direct IPS	1.05 (0.031)	1.95 (0.021)	1.36 (0.015)	*p* = 0.004
Distilled Water (Group 5/control)				
Herculite ^TM^ Xrv Ultra	0.32 (0.010)	0.47 (0.015)	0,65 (0.015)	
Estelite Ʃ Quick	0.31 (0.010)	0.44 (0.010)	0.56 (0.021)	
Z Hermack	0.24 (0.015)	0.44 (0.010)	0.54 (0.015)	
Versa Comp Sultan	0.58 (0.015)	0.58 (0.010)	0.80 (0.015)	
Empress^®^ Direct IPS	0.76 (0.010)	0.94 (0.015)	1.07 (0.010)	*p* = 0.008

**Table 2:** Details of composite resin materials and preventive mouthwash used in this study.

	**Preventive Mouthwash**		
**Name**	**Composition**	**Group**	**Manufacturer**
Flocare Gel	Stannous Fluoride (0.4%) as active ingredient	1	Dentamerica^®^ California, CA, USA
Pascal Gel	Topical Preventive Treatment Gel contains Acidulated phosphate fluoride	2	2929 NE Northup EWay, Believue, WA 98004, USA
Pro-Relief Mouthwash	Arginine 0.8%, Sorbitol, Propylene Glycol, Tetrapotassium Pyrophosphate, Hydrogenated Castor Oil, PVM/MA Copolymer, Sodium Fluoride (225 ppm), Menthol, Sodium Saccharin, Citric Acid	3	Colgate Palmolive, Bangkok 10110, Thailand
Plax Soin Mouthwash	Cetylpyridinium Chloride 0.075% *w*/*w*, Sodium Fluoride 0.05% *w*/*w*, Water Glycerin, Propylene Glycol, Sorbitol, Sodium Saccharin, Menthol, Methylparaben	4	Colgate Palmolive, Bangkok 10110, Thailand
	**Composite Resin Materials**		
**Materials**	**Type/Composition**	**Manufacturer**	
Herculite ^TM^ Xrv Ultra	Nano-hybrid-composite, ethoxylated Bisphenol A-dimethacrylate, 2,2-ethylenedioxydiethyl dimethacrylate, 3-Methacryloxypropyltrimethoxysilane, bisphenol A-glycidyl methacrylate (BIS-GMA)	Kerr italia, S.rl Via passanti, 332 1-84018 Scafati (SA), Italy	
Estelite Ʃ Quick	Resin-based Restorative Material. Silica-zirconia fillers (82% by weight; 71% by volume), -methylethylidene)bis[4,1-phenyleneoxy(2-hydroxy-3,1-propanediyl)] bismethacrylate (10%–30%), ethylenedioxydiethyl dimethacrylate (5%–10%)	Tokuyama Dental Corporation 38-9, Taitou 1-chome, Tokyo, Japan	
Z Hermack	Universal Micro-hybrid Resin-Based composite. Highly dispersed silica fillers (0.04 µm), Dimethacrylate resin (EBDADMA), Triethyleneglycol dimethacrylate (TEGDMA), Photo initiators, Titanium oxide	Zhermack, 10045021 Badia Polesine (RO), Italy	
Versa Comp Sultan	Universal-Hybrid Composite. Barium Boron Fluoro Alumino Silicate, Glass, Amorphous silica, Urethane Modified Bis-GMA dimethacrylate, Polymerizable, Dimethacrylate, (1-methylethylidene)Bis[4,1-phenyleneoxy(2-hydroxy-3,1-propanediyl)] bismethacrylate	Sultan Health Care, 1301 Smile Way, York, PA 17404, USA	
Empress^®^ Direct IPS	Direct Refill Enamel. Barium glass, ytterbium trifluoride, mixed oxide, silicon dioxide inorganic fillers (75–79 wt %; 52–59 vol % and size range of 40 nm and 3000 nm; mean particle size of 550 nm), dimethacrylates (20–21.5 wt %, opalescent shade 17 wt %)	Ivoclar vivadent, Bendererstrasse 29494 Schaan, Liechtenstein	

All restorative materials used in this study had shade A3.
